# Cellular Antioxidant, Anti-Inflammatory, and Antiproliferative Activities from the Flowers, Leaves and Fruits of *Gallesia integrifolia* Spreng Harms

**DOI:** 10.3390/molecules28145406

**Published:** 2023-07-14

**Authors:** Gabriela Catuzo Canônico Silva, Mariane de Almeida Machado, Karina Sakumoto, Rodrigo Sadao Inumaro, José Eduardo Gonçalves, Filipa Mandim, Josiana Vaz, Juliana Silveira do Valle, Maria Graciela Iecher Faria, Suelen Pereira Ruiz, Ranulfo Piau Junior, Daniela Dib Gonçalves, Zilda Cristiani Gazim

**Affiliations:** 1 Graduate Program in Biotechnology Applied to Agriculture, Universidade Paranaense, Umuarama 87502-210, Brazil; gabriela.canonico@edu.unipar.br (G.C.C.S.); jsvalle@prof.unipar.br (J.S.d.V.); gracielaiecher@prof.unipar.br (M.G.I.F.); suelenruiz@prof.unipar.br (S.P.R.); 2 Graduate Program in Animal Science with Emphasis on Bioactive Products, Universidade Paranaense, Umuarama 87502-210, Brazil; mariane.machado@edu.unipar.br (M.d.A.M.); piau@prof.unipar.br (R.P.J.); danieladib@prof.unipar.br (D.D.G.); 3 Graduate Program in Medicinal and Phytotherapeutic Plants in Primary Care, Universidade Paranaense, Umuarama 87502-210, Brazil; karina.sakumoto@edu.unipar.br; 4 Graduate Program in Clean Technologies, UniCesumar, Maringá 87050-390, Brazil; rodrigoinumaro@gmail.com (R.S.I.); jose.goncalves@unicesumar.edu.br (J.E.G.); 5 Cesumar Institute of Science, Technology and Innovation, UniCesumar, Maringá 87050-390, Brazil; 6 Centro de Investigação de Montanha (CIMO), Instituto Politécnico de Bragança, Campus de Santa Apolónia, 5300-253 Bragança, Portugal; filipamandim@ipb.pt (F.M.); josiana@ipb.pt (J.V.); 7 Laboratório Associado para a Sustentabilidade e Tecnologia em Regiões de Montanha (SusTEC), Instituto Politécnico de Bragança, Campus de Santa Apolónia, 5300-253 Bragança, Portugal

**Keywords:** pau d’alho, organosulfur, disulfide, 2,3,5-trithiahexane, lenthionine, triterpene glycosides, flavonoids

## Abstract

*Gallesia integrifolia,* a notable species in the Atlantic Forest, has been traditionally employed in folk medicine for treating rheumatism, asthma, and worms. This study investigated the cellular antioxidant, antiproliferative, and anti-inflammatory activities of the essential oils (EOs) and crude extracts (CEs) from *G. integrifolia* flowers, fruits, and leaves. The chemical identification of EOs was performed by GC–MS and CEs by UHPLC–MS. Cellular antioxidant and anti-inflammatory activities were assessed through mouse macrophage cell culture. In addition, the antiproliferative potential was evaluated in gastric, colorectal, breast, and lung tumor cell lines and non-tumor VERO cells. EOs predominantly contained organosulfur compounds in flowers (96.29%), fruits (94.94%), and leaves (90.72%). We found the main compound is 2,2′-Disulfanediyldiethanethiol in the EOs of flowers (47.00%), leaves (41.82%), and fruits (44.39%). Phenolic compounds were identified in CEs. The EOs and CEs demonstrated potential against the tumor cell lines tested (GI_50_ between 51 and 230 µg/mL). The selectivity index values were greater than 1.0 (1.01 to 3.37), suggesting a relative safety profile. Moreover, the anti-inflammatory activity IC_50_ ranged from 36.00 to 268 µg/mL, and the cellular oxidation inhibition ranged from 69% to 82%. The results suggest that oils and extracts derived from *G. integrifolia* have potential for use in various industrial sectors.

## 1. Introduction

Plants contain a variety of biologically active substances that exhibit a range of biological properties, including antioxidant activity. The ability of certain plants to counteract free radicals is due to the presence of numerous compounds, including phenolic compounds, tocopherols, carotenoids, ascorbic acid, peptides, and macromolecules such as polysaccharides [1].

Phenolic compounds are naturally present in plant tissues and constitute a diverse group of molecules characterized by distinct structures, chemical properties, and biological activities. The defining characteristic among these compounds is the presence of an aromatic ring, with either one hydroxyl group (simple phenolic compounds) or multiple hydroxyl groups (polyphenols), attached directly to a carbon atom in one (hydroxybenzoic and hydroxycinnamic acids) or more (flavonoids) benzene rings [2].

The antioxidant properties of phenolic compounds stem from combining the aromatic ring of their structure with substituents such as −OH or −OCH_3_. These substituents are recognized for their excellent ability to scavenge free radicals. Phenolic compounds directly intercept O_2_ and hinder lipid peroxidation by capturing lipid alkoxy radicals. Moreover, the antioxidant effects of phenols can be attributed to their capacity to alter the kinetics of peroxidation. Modifying the lipid package and reducing membrane fluidity, they restrict the diffusion ability of free radicals and mitigate the peroxidation process [3].

Flavonoids, primarily found in the glycosylated form, represent the most abundant category within the group of phenolic compounds. They are characterized by the presence of two aromatic rings connected by a three-carbon heterocyclic ring. Depending on the structural variations in the heterocyclic ring, these compounds can be further classified into various subgroups, including flavones, isoflavones, flavonols, flavanols, flavanones, anthocyanins, coumarins, and chalcones [4]. The antioxidant capacity of flavonoids involves multiple mechanisms, such as electron transfer to free radicals, inhibition of oxidase enzymes, reduction of alpha-tocopherol radicals, activation of antioxidant enzymes, chelation of metals, and their elimination from the reaction medium. Additionally, flavonoids can block the angiotensin-converting enzyme (ACE), which is associated with increased blood pressure; inhibit enzymes which are linked to estrogen and implicated in breast cancer; and suppress cyclooxygenase, a known source of prostaglandins [5].

Sulfur-containing compounds, such as thiols, disulfides, and sulfides, are recognized for their antioxidant properties. Among these, sulfides play a crucial role as antioxidants, facilitating the destruction of hydroperoxides without generating radical species. Additionally, sulfur-containing antioxidants can react with peroxyl radicals and are utilized as antioxidant additives in oils and polymers. The combination of a sterically hindered phenol and a sulfide group within a single molecule offers a unique approach to inhibit oxidative damage. In this combined delivery system, the hydroxy group of the phenolic moiety actively engages in reactions with peroxyl radicals, while the thioether group reacts with hydroperoxides, leading to the formation of sulfoxides (sulfones) and alcohols [6].

*Gallesia integrifolia* (Spreng) Harms, a member of the Phytolaccaceae family, is a species that remains relatively unexplored from both chemical and pharmacological perspectives. This species contains phenolic and thioethers compounds and holds significance as one of the prominent representatives of the Atlantic Forest [7]. Commonly referred to as garlic-wood, árbol de ajo, or pau d’alho, *G. integrifolia* emits a distinct garlic-like odor attributed to its essential oils, which possess a high concentration of sulfur compounds [8]. In traditional medicine, the leaves and bark of this plant are utilized for various purposes, including combating influenza, cough, pneumonia, prostate tumors, and rheumatism [9].

Studies conducted with essential oils and extracts from this species indicate anti-inflammatory, antiproliferative and antitumor [10], antifungal [8,11], acaricidal and larvicidal potential against *Rhipicephalus microplus* [12,13], larvicidal against *Aedes aegypt* [14], antiviral [15], antimycobacterial [9], and antiulcer [16] properties.

Phytochemical analysis of crude extracts from flowers and fruits of *G. integrifolia* indicated high concentrations of vitamin C and organosulfur compounds, and in leaves, high concentrations of phytol, fatty acids, and fatty acid esters [10]. Oxygenated diterpenes were also found in flowers and leaves, as well as phytosterol and triterpenes in fruits [13]. Organosulfur in essential oils represents 90% of all leaf, flower, and fruit compounds. In fruits, the main compounds were 2,3,5-trithiahexane, 3,6-dithiaoctan-1,8-diol, methanethiol, 2,8-dithianonane, dimethyl disulfide, and lenthionine [12]. The flowers have methionine ethyl ester, and the leaves have 3,5-dithiahexanol-5,5-dioxide [14]. Extracts prepared with the inner part of the stem bark have phenolic compounds such as gallic acid, rutin, and morin [17].

In this context, the objective of the present study was to investigate the antitumor, anti-inflammatory, antiproliferative, and cellular antioxidant activities of the essential oils and crude extract of the flowers, leaves, and fruits of *G*. *integrifolia*. 

## 2. Results and Discussion

### 2.1. Yield and Physical–Chemical Indices

The yield, refractive index, rotational power, and density of the essential oils of *G*. *integrifolia* extracted from flowers, leaves, and fruits are shown in Table 1. Possible adulteration of essential oils can be detected through physical–chemical properties. These tests are determined through standardized methods such as measuring the refractive index, density, optical rotation and determination of ester, acid, or carbonyl indices, among others [18]. The density of the essential oils of *G*. *integrifolia* found in the present study was greater than 1, a characteristic of the oil extracted from the different parts of pau d’alho, with no significant difference between the plant parts such as flowers, leaves, and fruits (Table 1). These values are in accordance with Raimundo et al. [12], who found densities of 1.48, 1.45, and 1.37 g/mL for oil in fruits, leaves, and flowers, respectively, and also with Bortolucci et al. [10], who found a density of 1.48 g/mL for the EO of the fruits. 

Regarding the refractive index (Table 1), there was no significant difference between the analyzed Eos, in line with Raimundo et al. [12], who found 1.6205 for fruits, 1.6075 for leaves, and 1.6224 for flowers, and with Bortolucci et al. [10], who found 1.620 for the EO of fruits. 

The parameters of yield and rotational power are directly related to abiotic factors, being influenced by seasonality. This difference can be observed when comparing the data from the present study with the yields obtained by Raimundo et al. [14] for flowers (0.19%), leaves (0.056%), and fruits (0.14%). Furthermore, a difference was also observed in the EO yield of the fruits (0.05%) found by Bortolucci et al. [10]. 

Regarding the specific optical rotation parameter (Table 1), there was a significant difference in the angle of deviation of the EOs. However, all the samples exhibited a right-handed deviation (+). These findings are the first report in the literature on determining the rotational power of the EO of *G*. *integrifolia*.

### 2.2. Chemical Composition of Essential Oils

**Figure 1:**
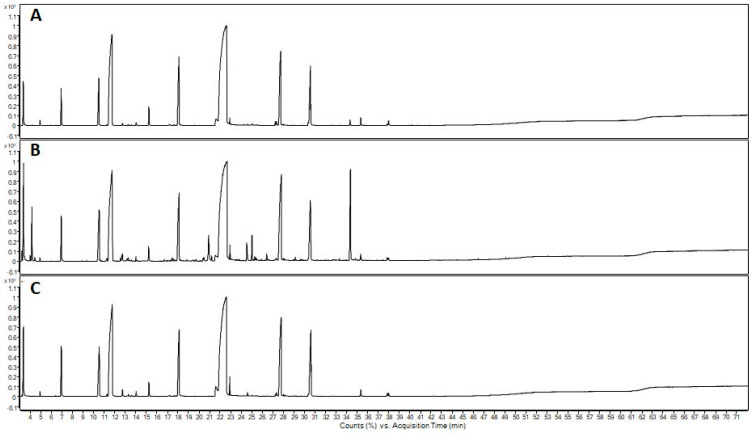
Chromatograms of essential oil from flowers (**A**), leaves (**B**), and fruits (**C**) of *Gallesia integrifolia* obtained by gas chromatography coupled to mass spectrometry (GC−MS).

**Figure 2:**
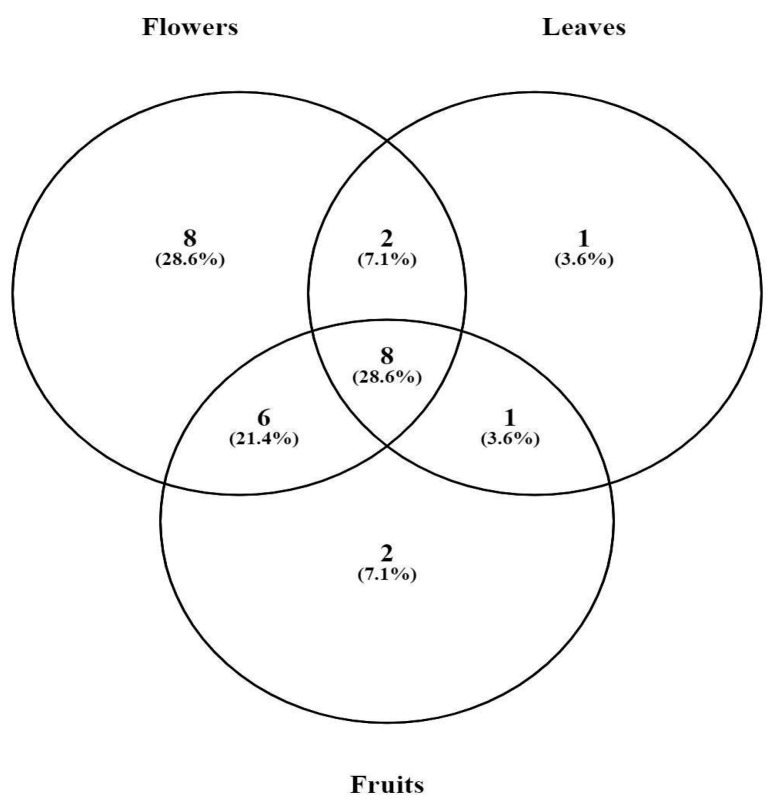
Venn diagram showing the distribution of compounds identified in *Gallesia integrifolia* leaves’, flowers’, and fruits’ essential oil. The Venn diagram was generated with the web tool provided by the Bioinformatics and Systems Biology of Gent (URL: https://bioinfogp.cnb.csic.es/tools/venny/ Accessed on 28 January 2023.).

The chemical analysis of the essential oils made it possible to identify the organosulfur compounds as the major class, finding 96.29% in the flowers, 90.72% in the leaves, and 94.94% in the fruits (Table 2 and Figure 1). Regarding the major compounds, the presence of 2,2′-Disulfanediyldiethanethiol was observed in the EO of flowers (47.00%), leaves (41.82%), and fruits (44.39%). The second most abundant compound was 2,3,5-trithiahexane in flowers (17.89%) and leaves (17.75%), followed by n-ethyl-1,3-dithioisoindole in flowers (13.94%), trithiomethoxymethane in leaves (15.04%), dimethyl trisulfide present in greater quantity in fruits (19.4%), and lenthionine in fruits (9.27%). Complementarily, a Venn diagram (Figure 2) was created to show the distribution of compounds identified in essential oils. Six compounds were present exclusively in flowers: (E)-hex-2-enal); nonanal; 5,6-di-hidro-2,4,6-trimetil-4H-1,3,5-ditiazina; 2,3,5,6-tetratiaheptane; 5-methyl-2-phenylindole; and 11,13-dihydroxy-tetradec-5-ynoic acid, methyl ester. Ethanol, 2-(octylthio) was the only compound present exclusively in the EO of the leaves. 3,6-Dithia-1,8-octanediol and hexathiepane were detected only in the fruits. Additionally, eight common elements occured in flowers, leaves, and fruits: dimethyl disulfide; 2,4-dithiapentane; dimethyl trisulfide; 1,2,4-trithiolane; 2,3,5-Trithiahexane; trithiomethoxymethane; lanthionine; and 2,2′-Disulfanediyldiethanethiol. Flowers and leaves shared two compounds: 1,3,5-trithiane and phytol. The EO of flowers and fruits had five compounds in common: linalool; dimethyl tetrasulfide; 1,2,4,5-tetrathiane; 3,5-dithiahexanol-5,5-dioxide; and N-ethyl-1,3-dithioisoindole. Only propane, 1,1′-thiobis[3-(methylthio) was found both in leaves and fruits. 

The chemical composition of essential oils (Table 2 and Figure 1) differed from those analyzed by Raimundo et al. [11,12,14] and Bortolucci et al. [10], who also investigated the essential oils of the flowers, leaves, and fruits of the same tree specimens used in this study. Differences may result from phenological changes and regional rainfall patterns observed over seven years of studies with this species. Regarding the phenological shifts in the present experiment, the flower buds were collected in March and the leaves in October 2020, while Raimundo et al. [12] collected floral buds and leaves in December 2015. That is, there was a difference of three months in the flowering period. This species fruits in the coldest months in the southern hemisphere (May, June, and July); in our experiment, the fruits were collected in July 2021, while Raimundo et al. [12] indicated that the collection occurred from May to July 2015.

During the collection period of plant parts, temperature and rainfall varied. During the gathering of flower buds, the temperature was 23 °C and there was 132 mm of rain. However, 23 °C and 94 mm were recorded when collecting fruits, and 23.8 °C and 195 mm when collecting leaves [19]. Raimundo et al. [12] collected flower buds and leaves at a temperature of 24.2 °C and 500 to 600 mm of rain, while the fruits were collected at 18.83 °C and 143.3 mm, indicating a change mainly in the flowering period and a difference in the rainfall regime in this region, with higher rainfall in 2015 than in 2021.

Therefore, differences in the chemical composition of the essential oils may have a direct relationship with the climatic changes of the last seven years and its influence on the phenology of the plant. According to Latocha et al. [20], genetic factors determine the concentration of bioactive compounds. However, external factors such as climatic conditions can affect the metabolic pathways, leading to the biosynthesis of different compounds.

**Table 2:** Chemical composition of *Gallesia integrifolia* flowers’, leaves’, and fruits’ essential oil by gas chromatography coupled to mass spectrometry (GC–MS).

Peak	RT	Compounds	MF	MW	Relative Area (%)			Retention Index (RI)		Source RI Literature
					Flowers	Leaves	Fruits	Calc.	Lit.	
1	3.337	dimethyl disulfide	C_2_H_6_S_2_	94	3.47	4.21	2.91	707	718	[21]
2	4.150	(E)-hex-2-enal	C_6_H_10_O	98	0.14	-	-	866	865	[22]
3	4.153	2,4-dithiapentane	C_3_H_8_S_2_	108	0.04	2.08	0.19	867	892	[23]
4	6.933	dimethyl trisulfide	C_2_H_6_S_3_	126	1.82	1.56	19.4	974	974	[24]
5	10.526	linalol	C_10_H_18_O	154	0.06	-	0.1	1015	1098	[25]
6	10.528	1,2,4-trithiolane	C_2_H_4_S_3_	124	2.31	3.59	3.86	1115	1127	[26]
7	10.704	Nonanal	C_9_H_18_O	142	0.07	-	-	1122	1112	[27]
8	11.272	n.i			-	-	0.18	1143		
9	11.284	2,3,5-Trithiahexane	C_3_H_8_S_3_	140	17.89	17.75	2.1	1143	1134	[28]
10	11.729	5,6-dihydro-2,4,6-trimethyl-4H-1,3,5-dithiazine	C_6_H_13_NS_2_	163	0.09	-	-	1158	1168	[29]
11	12.730	Tetrasulfide, dimethyl	C_2_H_6_S_4_	158	0.19	-	0.19	1291	1240	[30]
12	12.753	1,3,5-Trithiane	C_3_H_6_S_3_	138	0.14	5.4	-	1292	1271	[31]
13	13.227	n.i			0.23	-	0.56	1308		
14	13.350	2,3,5,6-tetrathiaheptane	C_3_H_8_S_4_	172	0.56	-	-	1313	1327	[32]
15	13.628	n.i			-	-	0.29	1324		
16	14.030	1,2,4,5-Tetrathiane	C_2_H_4_S_4_	156	0.05	-	8.30	1339	1337	[26]
17	15.276	Trithiomethoxymethane	C_4_H_10_S_3_	154	0.5	15.04	0.18	1383	1365	[33]
18	17.678	n.i			0.07	-	-	1473		
19	17.851	n.i			0.04	-	-	1479		
20	18.127	n.i			0.13	-	-	1489		
21	20.931	3,6-Dithia-1,8-octanediol	C_6_H_14_O_2_S_2_	182	-	-	0.31	1501	1503	[34]
22	20.946	n.i			0.16	-	-	1501		
23	22.461	Lenthionine	C_2_H_4_S_5_	188	0.34	0.75	9.27	1567	1590	[32]
24	24.551	3,5-dithiahexanol-5,5-dioxide	C_4_H_10_O_3_S_2_	170	6.17	-	0.06	1658	1633	[35]
25	25.037	n.i			0.11	0.79	0.24	1680		
26	27.216	2,2′-Disulfanediyldiethanethiol	C_4_H_10_S_4_	186	47.00	41.82	44.39	1781	1760	[10]
27	27.326	Ethanol, 2-(octylthio)	C_10_H_22_OS	190	-	1.57	-	1786	1792	[10]
28	27.381	n.i		207	0.21	-	-	1789		
29	28.071	hexathiepane	CH_2_S_6_	206	-	-	6.82	1922	1697	[36]
30	30.522	Phytol	C_20_H_40_O	296	0.18	4.6	-	2046	2027	[37]
31	30.559	N-ethyl-1,3-dithioisoindole	C_10_H_9_NS_2_	207	13.94	-	0.09	2047	2027	[11]
32	30.615	5 methyl-2 phenylindole	C_15_H_13_N	207	0.12	-	-	2150	2176	[11]
33	32.677	Propane, 1,1′-thiobis[3-(methylthio)	C_8_H_18_S_3_	210	3.75	0.54	0.17	2159	2193	[10]
34	37.887	11,13-Dihydroxy-tetradec-5-ynoic acid, methyl ester	C_15_H_26_O_4_	270	0.2	-	-	2359		[38]
35	37.964	n.i					0.19	2464		
36	62.623	n.i			-	-	0.19	3065		
		Total Identified			99.03	99.09	98.34			
		Organosulfur			96.02	90.72	94.38			
		Oxygenated monoterpenes			2.51	3.59	3.96			
		Diterpenes			0.50	4.60	-			
		Not identified			0.95	0.79	1.65			

The compounds are listed in elution order in HP-5MS UI column. The retention index (RI) was calculated using a standard homologous series of n-alkanes C7–C28 in the Agilent HP-5MS column. The identification was based on comparing mass spectra found in NIST 11.0 library. Relative area (%): percentage of the area occupied by the compound within the chromatogram; RI lit: Retention index found in the literature; n.i = Unidentified compounds; (-): Absent; RT: Retention time. PM: Molecular weight. FM: Molecular formula.

### 2.3. Chemical Composition of Crude Extracts

**Figure 3:**
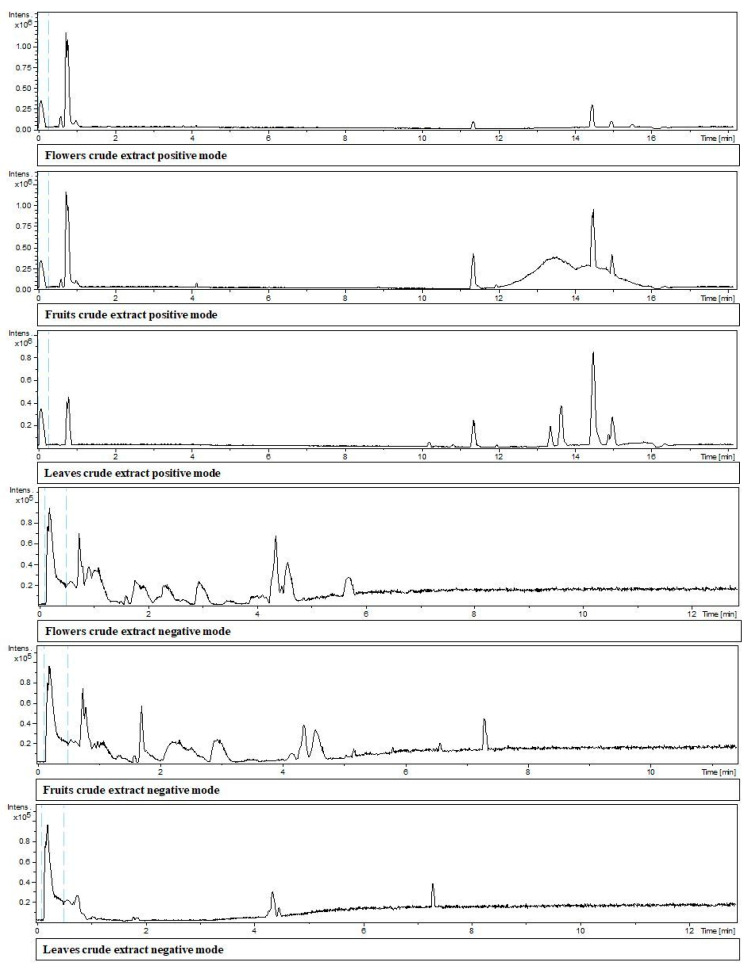
Chromatograms of *Gallesia integrifolia* flowers’, fruits’, and leaves’ crude extracts obtained by ultra-high-performance liquid chromatography coupled to high-resolution mass spectrometry (UHPLC–ESI–QTOF–MS/MS) analysis.

**Figure 4:**
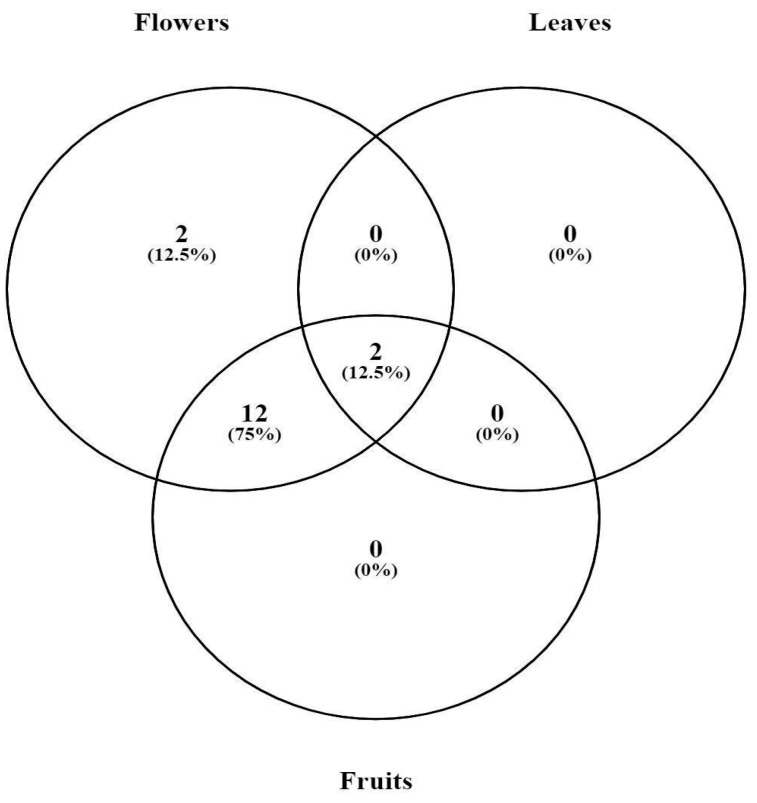
Venn diagram showing the distribution of compounds identified in *Gallesia integrifolia* leaves’, flowers’, and fruits’ crude extracts. The Venn diagram was generated with the web tool provided by the Bioinformatics and Systems Biology of Gent (URL: https://bioinfogp.cnb.csic.es/tools/venny/ Accessed on 28 January 2023).

The chemical identification of the crude extracts is listed in Table 3 and Figure 3, identified from the chromatograms obtained in both positive and negative mode (Figure 1). Complementarily, a Venn diagram (Figure 4) was created, showing the distribution of compounds identified in the crude extracts. For example, proline and quercimetrin were the only compounds present exclusively in flowers. On the other hand, esculentoside D and rutin were detected in flowers, leaves, and fruits. Additionally, 12 common compounds occurred in flowers and fruits: isoleucine; phytolaccoside E; phytolaccoside B; dipropyl disulfide; dibenzyl disulfide; kaempferol 5-glucoside; isorhamnetin 7- glucoside; kaempferol-3- O-glucoside-7-O-rhamnoside; kaempferol; quercetin; ascorbic acid; and 4-hydroxybenzoic acid.

The crude extracts presented a dark green color and a garlic odor, yielding 20.97% for flowers, 11.69% for leaves, and 15.08% for fruits. Regarding the chemical composition of the crude extracts identified by UHPLC (Table 3), it was possible to verify the presence of amino acids, triterpene saponins, triterpene glycosides, organosulfur compounds, flavonoids, vitamins, and organic acids. However, the compounds identified by Bortolucci et al. [13] in the crude extracts of *G*. *integrifolia* differ from those reported in this study, being alpha acids, fatty acids, fatty acid esters, oxygenated sesquiterpenes, oxygenated diterpenes, phytosterol, steroidal compounds, triterpenes, vitamin precursors, and other compounds. The difference in chemical composition is probably due to the tool used for chemical identification, since Bortolucci et al. [13] identified the extracts by gas chromatography coupled to mass spectrometry (GC–MS), while, in this study, the extracts were evaluated by high-performance liquid chromatography coupled to mass spectrometry (UHPLC–MS). Another factor that may explain the difference between the extracts is the phenological changes and changes in rainfall over the years, which were discussed in detail in the essential oil section. Finally, it is worth noting that the plant material collection occurred in the same location and from the same adult specimen of *G*. *integrifolia*, ruling out changes that happened by using another adult specimen from other areas.

### 2.4. Cellular Antioxidant Activity (CAA)

The results of the cellular antioxidant activity showed that all tested samples demonstrate the capacity to inhibit the reactive oxygen species formation, with the percentage of oxidation inhibition ranging from 69% to 82%. However, the essential oils from leaves and fruits and crude extracts from flowers and fruits showed the best results, as observed in Table 4.

Lei et al. [39] identified several sulfur compounds in fresh *Lentinula edodes* mushrooms, mainly thioethers such as 1,2,4-trithiolane, dimethyl disulfide, dimethyl trisulfide, and lenthionine. Sulfur compounds contribute to the scent of *L. edodes* and other appreciated fungi such as European *Tuber* truffles, where dimethyl disulfide is one of the truffles’ most important aroma markers [40]. As with several species of the genus *Allium*, Li et al. [41] found high contents of sulfurous compounds, mainly dimethyl trisulfide, in the essential oil of the flowers from *Allium tenuissimum*. Additionally, they reported an elevated radical-scavenging activity on DPPH (2,2-diphenyl-2-picrylhydrazyl), ABTS (2,2′-azino-bis(3-ethylbenzothiazoline-6-sulfonic acid)), and hydroxyl radical of the essential oil. Dimethyl disulfide and dimethyl trisulfide were identified in *Allium sativum* essential oil [42], which was able to scavenge 86.97% of DPPH free radicals. Morales-López et al. [43] found dimethyl trisulfide (65.43%) and dimethyl disulfide (19.29%) as major constituents of the essential oil of *Brassica oleracea* var. *capitata* f. *alba* and reported a potent TBARS (thiobarbituric acid reactive substance) inhibition effect of the essential oil (IC_50_ = 0.51 mg/L). These thioethers were also found in the essential oils of the leaves and fruits of *G. integrifolia*, which leads us to suggest that the thioethers contributed to the antioxidant potential found (Table 4), since more than 90% of the chemical composition of the essential oils were organosulfur compounds (Table 2).

Danyliuk et al. [44] claimed that several thioether derivatives with an incorporated thiazole nucleus exhibit antioxidant, antitumor, and antiviral activities. These sulfurous compounds exhibit antioxidant activity, as the thioether (sulfide) functional group reacts with hydrogen peroxide and superoxide, effectively scavenging reactive oxygen species (ROS) [45]. Currently, there is an insufficient amount of information regarding the biological potential of thioether. Our results on the cellular antioxidant activity of *G. integrifolia* essential oil, rich in thioether, bring new data about the antioxidant potential of these compounds. Further research should be conducted to isolate these compounds and deepen our understanding of their biological activities.

The extracts also exhibited high antioxidant potential (Table 4), emphasizing fruits and flowers. Analyzing the chemical composition of the extracts (Table 3), the presence of substances considered standard antioxidants used in several protocols, such as quercetin, rutin, kaempferol, kaempferol 5-glucoside, ascorbic acid, among others, was evident. Rutin and its aglycone quercetin are flavonoids that have been demonstrated to have anti-inflammatory, antioxidant, and antitumor properties in human glioblastoma cell lines [46].

Quercetin (3, 31, 41, 5, 7-pentahydroxyflavone) is considered a potent antioxidant, acting as an anticancer, anti-inflammatory, and antiviral agent due to its ability to eliminate free radicals and bind transition metal ions, thus allowing the inhibition of lipid peroxidation. By scavenging free radicals, quercetin decreases the action of transcription factors that generate pro-inflammatory cytokines, often found elevated in patients suffering from chronic inflammatory diseases [47]. Rutin (3,30,40,5,7-pentahydroxyflavone-3-rhamnoglycoside) is often used in patients with capillary fragility, varicose veins, and hematomas, but recent papers have shown an important antioxidant effect [48]. These authors demonstrated rutin’s protective effect on fibroblasts when subjected to UVA radiation. Reactive oxygen species were measured (%) as fluorescence intensity after administration of rutin (10 µM) and subsequent exposure to UVA radiation for 1 and 2 h. In cells without rutin, the increase in ROS was 20% after 1 h of exposure and 200% after 2 h, whereas, in cells pre-treated with rutin, transmission levels were similar to those in cells not exposed to radiation. The authors concluded that rutin could significantly reduce ROS, evidencing its antioxidant effect [48].

Kaempferol and its glycosides kaempferol 5-glucoside and kaempferol-3-O-glucoside-7-O-rhamnoside (Table 3) function as antioxidants by eliminating ROS and preventing DNA damage. ROS reduction occurs by inducing phase 2 enzymes and altering signal transduction pathways. These flavonoids have been shown to inhibit the NF-κB transcription factor, stimulating the Nrf2 transcriptional pathway and restoring redox homeostasis in cells, contributing to cancer prevention through their antioxidant and anti-inflammatory effects [49]. Studies conducted by Kluska et al. [50] investigated the impact of kaempferol and its glycoside derivatives isolated from *Lens culinaris* Medik in HL-60 cells (human acute promyelocytic leukemia) and PBMCs (peripheral blood mononuclear cells) treated with etoposide. The authors demonstrated that kaempferol glycosides reduced etoposide-induced DNA damage in PBMCs. These authors suggested that the antioxidant activity of kaempferol is related to the activation of antioxidant genes and proteins [50].

Ascorbic acid is a potent antioxidant that neutralizes oxidative stress through an electron donation/transfer. Vitamin C reduces unstable species of oxygen, nitrogen, and sulfur and regenerates other antioxidants in the body, such as alpha-tocopherol (vitamin E). Furthermore, studies with human plasma have shown that vitamin C effectively prevents lipid peroxidation induced by peroxide radicals [51].

The cellular antioxidant activity assay selected in this research measures oxidation inhibition induced by peroxyl radicals of dichlorofluorescein by antioxidant substances in cell culture. This method has been developed in response to the need for a cell culture model to assess potential antioxidant capacities in vitro, because these are biologically more relevant than chemical antioxidant assays [52]. However, assays performed in vitro do not necessarily reflect cellular physiological conditions and do not consider bioavailability and metabolism issues. Furthermore, the mechanisms of action of antioxidants go beyond free radical scavenging antioxidant activity in disease prevention and health promotion. On the other hand, animal models and human studies are expensive and inadequate when aiming to perform an initial antioxidant screening of biomolecules, foods, and supplements. Therefore, there is a need to deploy cell culture models to support antioxidant research before animal studies and human clinical trials [53].

### 2.5. Antiproliferative and Anti-Inflammatory Activity

The results of the antiproliferative activity are shown in Table 5. Essential oils and crude extracts showed the capacity to inhibit the proliferation of the cell lines tested. The samples exhibited GI_50_ values between 51 and 230 µg/mL (Table 5). In general, the crude extracts exhibited higher potential, with lower GI_50_ values when compared with the essential oils. The colorectal adenocarcinoma (CaCo2) cancer cell line demonstrated higher susceptibility for the samples studied, while gastric adenocarcinoma (AGS) showed lower susceptibility with higher GI_50_ values. Although the samples demonstrated toxicity for the non-tumor line tested (VERO), the GI_50_ values were overall higher than those obtained for the tumor cell lines, providing a margin of safety.

The presence of flavonoids in crude extracts may explain the antiproliferative effect in the present study (Table 5). Based on the structure–activity relationship, the pharmacological effects of flavonoids mainly involve the regulation of macrophage recruitment, reprogramming, and activity. Sun et al. [54] investigated how flavonoids regulate tumor-associated macrophages (TAMs). Flavonoids play an essential role in the regulation of TAMs by binding to macrophages and cytokines on the surface of proteins. According to the authors, the number of hydroxyls, the C2=C3 double bond, and the structure similar to tyrosine kinase inhibitors determine that flavonoids can inhibit macrophage recruitment, inhibiting the chemokine ligand–motif chemokine receptor (CCL2-CCR2), inhibiting the release of macrophage colony-stimulating factor (M-CSF), and negatively regulating the expression of vascular endothelial growth factor (VEGF). The hydroxyl groups, carbonyl functional groups, methoxy groups, and catechol groups allow flavonoids to upregulate the expression of pro-inflammatory M1 macrophages, reduce the secretion of tumor-associated immunosuppressive cytokines, and remodel the macrophage phenotype. Finally, the C-ring state and estrogen-like chemical structure allow flavonoids to maintain the structural integrity and functional activity of macrophages by regulating the balance between oxides, increasing the level of macrophage phagocytosis, and reversing immunosuppression [54].

Kaempferol and quercetin belong to the flavan group and have the same benzene ring hydroxylation pattern. This pattern is essential for the ability of flavonoids to exert an antioxidant action. When flavonoids are exposed directly to ROS, they generally suffer a partial or total loss of their antioxidant properties, especially their ROS elimination/reduction property. However, this assumption was contested when alkalis oxidated flavonoids and the mixtures of metabolites formed exhibited antioxidant potential that, in some cases, were equal to or superior to those of their precursors. Of particular interest was the case of quercetin, whose alkali-induced oxidation generated a mixture of metabolites, of which the main one was 2-(3,4-dihydroxybenzoyl)-2,4,6-trihydroxy-3(2H)-benzofuranone. Isolation and testing of this metabolite in CaCo2 cell lines exposed to different ROS demonstrated that this metabolite offers complete antioxidant protection [55]. This information leads us to consider that quercetin and kaempferol identified in the *G. integrifolia* fruit extract (Table 3) may have contributed to the antiproliferative potential of the CaCo2 strain with GI_50_ 83.00 µg/mL (Table 5).

Vitamin C has a role in the epigenetic regulation of gene expression, functioning as a cofactor for the translocation enzymes family, which catalyze the removal of DNA methylated cytosine (5 mC) through its hydroxylation to 5-hydroxymethylcytosine (5 hmC). The 5 hmC is a DNA demethylation intermediate and an epigenetic marker acting as a transcriptional regulator. Acquired epigenetic changes are expected during the development and progression of many cancers. Reduction of 5 hmC due to the down-regulation of translocation family enzymes represents a characteristic feature of cancers. Treatment with vitamin C has been shown to increase the content of 5 hmC in cancer cell lines, causing a consequent change in the transcriptome and a decrease in the malignant phenotype [51,56].

The selectivity index (SI) of the samples was also determined. The essential oils and crude extracts presented SI > 1.0 with values between 1.01 and 3.37, indicating low cytotoxicity (Table 5). Nevertheless, the EO and EB of the leaves tested against the cell line NCI–H460 exhibited SI values of 0.95 and 0.77, respectively. Similarly, the EO and EB of the flowers evaluated against the cell line MCF-7 presented SIs of 0.92 and 0.99, respectively, showing a high cytotoxic potential for these two cell lines. Bortolucci et al. [10] also investigated the cytotoxicity of the essential oil of the fruits of *G. integrifolia*, finding SIs ranging from 1.67 to 6.06, which indicates high selectivity for tumor cells. Furthermore, studies conducted by Bortolucci et al. [10] found that the essential oil of the fruits tested in the same cell lineage of this experiment presented GI_50_ ranging from 66.00 to >400 µg/mL, reinforcing the low antitumor potential against the tested cells.

The studied essential oils and crude extracts showed anti-inflammatory potential, with IC_50_ ranging from 36.00 to 268 µg/mL compared to the positive control dexamethasone (EC_50_ = 6.30 µg/mL) (Table 6). A comparative analysis showed that the essential oil of the fruits presented better results with EC_50_ = 36.00 µg/mL, followed by the EO of the leaves with EC_50_ = 54.00 µg/mL. These values were only 5.71 and 8.57 times less active than the dexamethasone positive control (EC_50_ = 6.3 µg/mL). Bortolucci et al. [10] also reported the anti-inflammatory potential of the EO from fruits (EC_50_ = 55 µg/mL) collected from the same specimen of *G. integrifolia* used in our study.

The presence of lenthionine (9.27%) and dimethyl trisulfide (19.40%) was observed in the EO of the fruits (Table 3). Bortolucci et al. [10] also found these compounds in the EO of fruits (Table 7). We suggest that these two compounds may be related to the observed anti-inflammatory potential, since lenthionine has great anti-inflammatory activity and is even capable of reducing the production of TNF-α (tumor necrosis factor). This hypothesis can be corroborated by studies conducted by Kupcova et al. [57], who investigated the ability of lenthionine to reduce TNF-α production at a concentration of 1.0 µg/mL. Its effect was greater than that of the reference drug prednisone, a synthetic corticosteroid used as an immunosuppressant.

Dimethyl trisulfide, another compound possessing anti-inflammatory properties, was identified in the essential oil of fruits and flowers of *G. integrifolia.* Dombi et al. [58] demonstrated that dimethyl trisulfide provides relief of neuropathic pain induced by partial sciatic nerve ligation in mice. Notably, the beneficial effect of dimethyl trisulfide was mediated through TRPA1 and SST4 somatostatin receptors, indicating that this compound could become a complementary treatment option for neuropathic pain.

Thioethers’ anti-inflammatory action was verified in studies by Tarudji et al. [45]. The authors used cross-linked thioether core nanoparticles (thioether-NP) to reduce secondary lesion spread in a mouse model of mild controlled cortical injury. The administration of thioether-NP led to a reduction in various indicators of neuroinflammation, such as the density of activated microglia and the formation of neuron–astrocyte–microglia triads in the adjacent healthy brain tissue. The author attributed these positive outcomes to the remarkable ROS scavenging ability of thioether-NP.

The anti-inflammatory action of the crude extracts of flowers EC_50_ = 51.00 µg/mL and fruits EC_50_ = 48.00 µg/mL is worth mentioning. These results can be explained due to the presence of flavonoids, particularly quercetin, in these extracts (Table 3). In plants, flavonoids protect against ultraviolet rays, insects, and fungi. Among quercetin’s pharmacological activities are reports of antitumor, anti-inflammatory, antioxidant, and antiviral activity, as well as hormonal regulation [59]. A study conducted by Ozyel et al. [60] explored the potential of quercetin in reducing the harmful effects of hyperglycemia and inflammation on vascular endothelium. Using human umbilical vein endothelial cells (HUVECs) exposed to high glucose levels, the researchers identified and measured metabolites. The results showed that quercetin was effective in shifting the balance of HUVEC metabolites towards a less inflamed state, even when exposed to pro-inflammatory stimuli.

Overall, *G. integrifolia* essential oils and extracts containing sulfur and phenolic compounds have been found to possess the ability to inhibit the formation of reactive oxygen species. Moreover, they exhibit the potential to hinder the growth of tested cell lines, with extracts displaying greater potential. Additionally, essential oils derived from leaves and fruits have higher anti-inflammatory activity.

Our research aims to shed more light on the species *G. integrifolia*, highlighting the potential benefits of its leaves, flowers, and fruits, which are often overlooked and considered waste. Traditional medicine mainly focuses on the tree’s bark, but we believe that exploring the chemical composition and conducting in vivo studies will confirm the bioactivity results obtained in this study. Further research is necessary to fully understand the potential of this species.

**Table 7:** Chemical composition of *Gallesia integrifolia* essential oil of leaves, flowers, and fruits obtained by different authors.

Localization	Extraction Method/Time	Rainfall Index	Collection Period	Plant Part			Source
				Flowers	Leaves	Fruits	
Umuarama, Brazil S 23°46′16″ WO 53°19′38″ 442 m altitude	Hydrodistillation (3 h)	132 mm flowers (March) 94 mm fruits (July) 195 mm leaves (October)	Flowers (March 2021) Fruits (July 2021) Leaves (October 2021)	2,2′-Disulfanediyldiethanethiol (47.00%) 2,3,5-Trithiahexane (17.89%) n-ethyl-1,3-dithioisoindole (13.94%)	2,2′-Disulfanediyldiethanethiol (41.82%) 2,3,5-Trithiahexane (17.75%) trithiomethoxymethane (15.04%)	2,2′-Disulfanediyldiethanethiol (44.39%) dimethyl trisulfide (19.40%) lenthionine (9.27%)	Current study
Umuarama, Brazil S 23°46′16″ WO 53°19′38″ 442 m altitude	Hydrodistillation (3 h)	500–600 mm leaves and flowers (December) 143.3 mm fruits (June)	Flowers (December 2015) Leaves (December 2015) Fruits (May/June 2015)	disulfide, dimethyl (43.72%) methanethiol (44.91%)	butanal, 3-metil (40.43%) disulfide, dimethyl (40.42%) α-Terpinolene (8.63%)	2,3,5-Trithiahexane (35.29%) 3,6-dithiaoctan-1,8-diol (20.89%) Methanethiol (16.26%)	[12]
Umuarama, Brazil S 23°46′16″ WO 53°19′38″ 442 m altitude	Hydrodistillation (3 h)	143.3 mm fruits (June)	Fruits (May/June 2015)			dimethyl trisulfide (15.49%) 2,8-dithianonane (52.63%) lenthionine (14.69%)	[11]
Umuarama, Brazil S 23°46′16″ WO 53°19′38″ 442 m altitude	Hydrodistillation (3 h)	500–600 mm flowers and leaves (December) 143.3 mm fruits (June)	Flowers (December 2015) Leaves (December 2015) Fruits (May/June 2015)	methionine, ethyl ester (45.28%) methyl p-tolyl sulfide (17.08%) n-ethyl-1,3-dithioisoindole (13.40%)	3,5-dithiahexanol-5,5-dioxide (38.93%) 1,3,5-trithiane (13.74%) n-ethyl-1,3-dithioisoindole (12.58%)	dimethyl trisulfide (15.28%) 2,8dithianonane (52.63%) Lenthionine (14.69%)	[14]
Umuarama, Brazil S 23o46′16″ WO 53°19′38″ 442 m altitude	Hydrodistillation (3 h)	143.3 mm fruits (June)	Fruits (May/June 2015)			dimethyl trisulfide (15.15%) 2,8 dithianonane (52.86%) lenthionine (14.75%)	[61]

## 3. Materials and Methods

### 3.1. Plant Material

The plant material was collected in the municipality of Umuarama-Paraná, Brazil, at latitude 23°46′16″ S, longitude 53°19′38″ W, and altitude of 442 m. The flowers of *Gallesia integrifolia* were collected in March and April, the fruits in June, and the leaves in October 2020 (after the fruiting period). A voucher specimen was deposited in the Herbarium of the State University of Western Paraná (UNIOESTE) under number 1716. This species is registered in the National System for the Management of Genetic Heritage and Associated Traditional Knowledge (SisGen) under number A4C370B.

### 3.2. Essential Oil Extraction and Preparation of Crude Extract from the Flowers, Leaves, and Fruits of Gallesia integrifolia

Flowers, fresh fruits, and leaves were used separately to extract the essential oil. They were fragmented in a crusher for 5 min with distilled water and submitted to hydrodistillation in a modified Clevenger apparatus for 3 h [11]. At the end of the distillation, the essential oil was removed with hexane P.A. using a glass Pasteur pipette, filtered with anhydrous sodium sulfate, transferred to amber vials, and stored at −4 °C for solvent evaporation. The yield (%) of essential oils was calculated from the ratio between the mass (g) of dry plant material and the mass (g) of oil obtained, expressed in percentage. The extractions were performed in triplicate.

### 3.3. Obtaining the Crude Extract of the Leaves, Flowers, and Fruits of Gallesia integrifolia

For the preparation of the extracts, flowers (247.50 g), fruits (314 g), and leaves (360 g) were previously dried at room temperature. The plant material was fragmented to a granulometry of 710 µm for leaves and fruits and 850 µm for flowers, and submitted to dynamic maceration, with renewal of the solvent (ethyl alcohol 96°GL), until the plant material was utterly exhausted. Then, the filtrate was concentrated under reduced pressure in a rotary evaporator (Tecnal TE-210) at 40 °C until obtaining the crude extracts. The yield (%) of the extracts was calculated from the ratio between the mass (g) of the dry plant material and the mass (g) of the obtained extract, expressed in percentage [61].

### 3.4. Physicochemical Index of Essential oils

#### 3.4.1. Refractive Index

The refractive index was determined as a function of sodium light at a wavelength of 589.3 nm (ray D) at 20 ± 0.5 °C. This analysis was determined in an ABBE refractometer, model RL3, brand PZO Warszawa, at 20 °C, and the value was expressed as $n_{D}^{20}$, according to the Brazilian Pharmacopoeia [62].

#### 3.4.2. Specific Optical Rotation

The specific optical rotation was calculated from the optical rotation observed in the essential oil solution, using an ACA TEC automated polarimeter. The solvent used for the dilution of the Eos was absolute alcohol. The concentrations of Eos used in this test were 0.49% for flowers and leaves and 0.37% for fruits. Optical rotation measurements are performed at 598 nm at 20 °C, using a 10 cm tube. The specific optical rotation was calculated according to the formula described in the Brazilian Pharmacopoeia [62], and the value was expressed as ${\left|\alpha \right|}_{D}^{20}$.

#### 3.4.3. Absolute Density

The density (*d*) was determined by capillarimetry, using a 5 µL graduated glass capillary, determining the ratio between the mass (g) and the volume (mL) of essential oil inside the capillary, at 20 °C, and was expressed as $d_{20}^{20}$ [62].

### 3.5. Chemical Identification of Essential Oils by Gas Chromatography Coupled to Mass Spectrometry (GC–MS)

The essential oil samples were diluted in a 1:10 ratio with dichloromethane and subjected to analysis using gas chromatography coupled with mass spectrometry (GC–MS) on an Agilent 7890B-5977A MSD instrument. The analysis utilized an HP-5MS UI 5% (30 m × 0.25 mm × 0.25 µm) capillary column with specific temperature parameters. Initially, the oven temperature was set at 60 °C for 3 min. Subsequently, the temperature was gradually increased at a rate of 5 °C/min until it reached 300 °C, where it was maintained for 10 min. Finally, the temperature was further increased to 310 °C at a rate of 10 °C/min for an additional 10 min. The carrier gas employed was helium, flowing at a linear speed of 1 mL/min. For injection, the essential oil samples were introduced in split mode (20:1), with an injection volume of 1 µL at an injector temperature of 300 °C. The transfer line, ionization source, and quadrupole were maintained at temperatures of 280 °C, 230 °C, and 150 °C, respectively. Mass spectrometry data were acquired within a scan range of 30 to 550 *m/z,* with a solvent delay of 3 min. The identification of compounds was accomplished by comparing their mass spectra with those available in the NIST 11.0 libraries, in addition to evaluating their retention indices (RI) obtained using a standard homologous series (C_7_–C_40_) [63].

### 3.6. Chemical Identification of Crude Extracts by Ultra-High-Performance Liquid Chromatography Coupled to High-Resolution Mass Spectrometry (UHPLC-ESI–QTOF-MS/MS)

To chemically characterize the samples, 1.0 mg of the crude extracts was dissolved in 1.0 mL of methanol and subjected to analysis using ultra-high-performance liquid chromatography (Nexera X2, Shimadzu, Kyoto, Japan), coupled with a high-resolution mass spectrometer (QTOF Impact II, Bruker Daltonics Corporation, Drive Billerica, MA, USA) equipped with an electrospray ionization source. The capillary voltage was set to operate in both positive and negative ionization mode at 4500 V with an end-plate potential of −500 V. Dry gas parameters were adjusted to a flow rate of 8 L/min and a temperature of 200 °C, while the nebulization gas pressure was maintained at 4 bar. For collision-induced dissociation, argon gas was employed, with a collision energy ranging from 15 to 30 eV. Data acquisition occurred within the 50–1300 *m/z* range, with a rate of five spectra per second. The selection of ions of interest was accomplished through automatic fragmentation of tandem mass spectrometry (MS/MS) data. Chromatographic separation was achieved using a C18 column (75 × 2.0 mm i.d., 1.6 µm Shim-pack XR-ODS III) and a gradient mixture of solvents A (H_2_O) and B (acetonitrile). The gradient elution profile was as follows: starting with 5% B from 0 to 1 min, increasing to 30% B from 1 to 4 min, further increasing to 95% B from 4 to 8 min, and maintaining at 95% B from 8 to 17 min, all at a column temperature of 40 °C. For compound identification, the proposed approach followed review studies on the genus Phytolaccaceae, utilizing mass error calculations and comparison of results with data from MassBank (http://www.massbank.jp/ Accessed on 28 January 2023) and the Human Metabolome Database (http://www.hmdb.ca/ Accessed on 28 January 2023).

### 3.7. Cellular Antioxidant Activity

The extract samples were dissolved in water, while the essential oils were dissolved in a mixture of DMSO and water (50:50, *v/v*) to achieve a concentration of 8 mg/mL. To prepare the samples for testing, all samples were diluted with 2′,7′-dichlorohydrofluorescein (DCFH) prepared in ethanol and further diluted with HBSS to a final concentration of 50 μM. The concentrations to be tested ranged from 500 to 2000 μg/mL.

This procedure was conducted following the protocol outlined by de La Fuente et al. [64]. The cell line used was RAW 246.7 mouse macrophages (acquired from DMSMZ-Leibniz-Institut DSMZ-Deutsche Sammlung von Mikroorganismen und Zellkulturen GmbH, Braunschweig, German). The cells were maintained in DMEM culture medium supplemented with heat-inactivated fetal bovine serum (SFB) at a concentration of 10%, along with penicillin (100 U/mL), streptomycin (100 mg/mL), and L-glutamine (2 mM). The culture flasks were kept inside an incubator set at 37 °C, with 5% CO_2_ and a humid atmosphere. The cells were utilized when they reached a confluence of 70 to 80%.

Mouse macrophages were detached, and a solution containing a cell density of 70,000 cells per mL was prepared using the ScepterTM Handheld Automated Cell Counter from Millipore Corporation in Billerica, MA, USA. A volume of 300 μL from the prepared solution was transferred to black 96-well microplates with clear bottoms manufactured by SPL Lifesciences. The microplates were then incubated for 48 h.

Following the incubation period, the culture medium was removed, and the cells were rinsed with HBSS twice using 100 μL each time. Subsequently, the cells were exposed to the desired concentrations of the extracts (200 μL; 500–2000 μM) and incubated for 1 h. After the incubation, the cells were washed with HBSS twice using 100 μL each time, and a solution of 2.2 2′-azobis(2-methylpropionamide) dihydrochloride (AAPH) was added (100 μL; 600 μM). Fluorescence readings were taken every 5 min for a total of 1 h (Synergy H1, BioTek Instruments, Winooski, VT, USA) with excitation at 485 nm and emission at 538 nm. Quercetin was used as a positive control, and the dichlorohydroflurescein and DMEM culture medium were used as a negative control. The results were expressed as the percentage of inhibition observed at the highest concentration tested (2 mg/mL) [64].

### 3.8. Antiproliferative Activity in Human Tumor and Non-Tumor Cell Lines

Various human tumor cell lines were included in this study: AGS (gastric adenocarcinoma), CaCo-2 (colorectal adenocarcinoma) (acquired from European Collection of Authenticated Cell Cultures—ECACC), MCF7 (breast adenocarcinoma), NCI-H460 (lung carcinoma) (acquired from Leibniz—Institute DSMZ). Additionally, the non-tumor cell line VERO (African green monkey kidney) (acquired from the European Collection of Authenticated Cell Cultures—ECACC) was also tested. All cell lines were cultured in RPMI-1640 medium supplemented with 10% fetal bovine serum, 2 mM glutamine, 100 U/mL penicillin, and 100 mg/mL streptomycin, except for VERO, which was maintained in high glucose DMEM medium, as previously described in Section 3.7.

To prepare the extracts, they were dissolved in sterile water, while the essential oils were dissolved in a mixture of DMSO and water (50:50, *v/v*) to achieve a concentration of 8 mg/mL. From this stock solution, successive dilutions ranging from 0.125 to 8 mg/mL were prepared. Each extract concentration (10 μL) was incubated with the cell suspension (190 μL) of the respective cell lines in 96-well microplates for 72 h. The final concentrations tested ranged from 6.25 to 400 µg/mL. The microplates were incubated under conditions of 37 °C, 5% CO_2_, and a humid atmosphere, after confirming cell adherence. All cell lines, except VERO, were seeded at a density of 10,000 cells per well, while VERO utilized a density of 19,000 cells per well. After the incubation period, the cells were treated as follows. TCA (10% *w/v*; 100 μL), previously cooled, was added and the plates were incubated for 1 h at 4 °C. The plates were then washed with water and, once dry, an SRB solution (0.057% *w/v*; 100 μL) was added and allowed to stand at room temperature for 30 min. To remove non-adhered SRB, plates were washed three times with 1% acetic acid (*v/v*) and left to dry. Finally, the adhered SRB was solubilized with Tris (10 mM, 200 μL), and the absorbance at 540 nm was measured using a microplate reader (Synergy H1, BioTek Instruments, Winooski, Vermont, USA). The results were expressed as the concentration of the extract required to inhibit cell growth by 50% (GI_50_). Ellipticine was used as a positive control [65]. The selectivity index (SI) was calculated as the ratio between the GI_50_ values for VERO cells and the tumor cells.

### 3.9. Anti-Inflammatory Activity of Gallesia Integrifolia Flowers’, Fruits’, and Leave’s Essential Oils and Crude Extracts

To achieve a final concentration of 8 mg/mL, the extracts and essential oils were dissolved in water or DMSO and water (50:50, *v/v*) respectively. Dilutions were then prepared, resulting in the desired concentrations for testing (0.125–8 mg/mL). The RAW 264.7 mouse macrophage cell line (acquired from DMSMZ-Leibniz-Institut DSMZ-Deutsche Sammlung von Mikroorganismen und Zellkulturen GmbH) was cultured in high-glucose DMEM medium, supplemented according to the previously mentioned conditions (Section 3.7). The cells were detached using a cell scraper. Next, an aliquot of the macrophage cell suspension (300 μL) with a cell density of 5 × 10^5^ cells per mL was transferred to 96-well plates. The microplate was then incubated under the previously specified conditions for 24 h to allow proper cell adhesion and growth. After this period, the cells were treated with different concentrations of the extract (15 μL, 0.125–8 mg/mL) and incubated for one hour. Stimulation was achieved by adding 30 μL of a lipopolysaccharide (LPS) solution (1 mg/mL) and adjusting the volume to 300 μL. The plates were further incubated for an additional 24 h (final concentrations tested 6.25–400 μg/mL). As a positive control, dexamethasone (50 mM) was used, while negative controls consisted of cells in the absence of treatment and cells exposed to LPS. Nitric oxide levels were measured using a Griess reagent system kit (nitrophenamide, ethylenediamine, and nitrite solutions; Catalog No. 328670500, Thermo Fisher Scientific India Pvt. Ltd. Waltham, Massachusetts, USA). A nitrite calibration curve (concentrations ranging from 100 to 0.78125 µM sodium nitrite at 1.6 mM) was prepared in a 96-well plate (y = 0.0068x + 0.0951, R^2^ = 0.9864). The absorbance at 540 nm was measured using a microplate reader (Synergy H1, BioTek Instruments, Winooski, Vermont, USA) to determine the amount of nitric oxide produced. Nitric oxide concentrations were determined by extrapolating from the standard curve. The percentage of inhibition compared to the negative control values was calculated based on the extracts’ concentrations that resulted in 50% inhibition of nitric oxide production, using mathematical regression [65].

### 3.10. Statistical Analysis

The results of cellular antioxidant, anti-proliferative, and anti-inflammatory activities were expressed as mean ± standard deviation. Data were submitted to analysis of variance (ANOVA) and compared by Tukey test (*p* ≤ 0.05) utilizing SPSS statistics 22 software. StatSoft Statistics 10.0, South America, 2022.

## 4. Conclusions

This study unveils novel insights into the chemical composition and health-promoting properties of essential oils and extracts derived from the leaves, flowers, and fruits of *Gallesia integrifolia*. The essential oils are rich in thioether compounds, while the crude extracts contain phenolic compounds, exhibiting significant antioxidant potential and antiproliferative effects in cell cultures. Notably, the extracts derived from the flowers and fruits display enhanced anti-inflammatory properties. These preliminary findings underscore the considerable potential of *G. integrifolia* as a valuable source of phenolic compounds and thioether. Consequently, it is plausible to hypothesize that the leaves, flowers, and fruits of *G. integrifolia* could find applications as natural remedies in food supplements, cosmetic formulations, and pharmaceutical products for the prevention and treatment of various diseases.

It is important to consider the value of the species being studied. Utilizing parts of the plant that are typically overlooked and thrown away contributes to research, promoting environmental sustainability and a circular economy.

## Figures and Tables

**Table 1:** Physicochemical indices for absolute density (g/mL), refractive index, specific optical rotation, and yield (%) of *Gallesia integrifolia* flowers’, fruits’, and leaves’ essential oil.

Samples	Refractive Index	Specific Optical Rotation	Absolute Density (g/mL)	Yield (%)
Flowers	1.6169 ± 0.0006 a	+8.28° ± 0.06 c	1.30 ± 0.05 a	0.452 ± 0.062 a
Fruits	1.6190 ± 0.01 a	+11.16° ± 0.1 b	1.15 ± 0.13 a	0.190 ± 0.023 b
Leaves	1.6075 ± 0.001 a	+12.65° ± 0.07 a	1.12 ± 0.02 a	0.049 ± 0.003 c

Values represent mean ± standard deviation and confidence intervals (CI) of essential oils (EO). Means followed by the same letters in the same column do not statistically differ according to Tukey’s test at the significance level of *p* < 0.05.

**Table 3:** Chemical composition of *Gallesia integrifolia* flowers’, leaves’, and fruits’ crude extracts by UHPLC-ESI–QTOF-MS/MS.

Compounds	Theoretical Mass *m/z*	Experimental Mass *m/z*	RT (min)	Error (ppm)	Sample
Proline	116.1011 [M+H]	116.0703	0.78	2.58	Flowers
Isoleucine	132.1019 [M+H]	132.1015	0.94	3.03	Flowers
		132.1014	0.93	3.04	Fruits
Esculentoside D	695.4001 [M+H]	695.3998	5.45	0.43	Flowers
		695.3992	5.40	1.29	Fruits
		695.3989	5.50	1.72	Leaves
Phytolaccoside E	865.3982 [M+K]	865.3608	4.04	43.21	Flowers
		865.3616	4.08	43.19	Fruits
Phytolaccoside B	703.3454 [M+K]	703.3292	3.66	23.03	Flowers
		703.3305	3.70	23.00	Fruits
Dipropyl disulfide	149.0453 [M−H]	149.0450	0.86	2.01	Flowers
		149.0452	0.87	0.67	Fruits
Dibenzyl disulfide	245.0453 [M−H]	245.0514	1.51	24.89	Flowers
		245.0514	1.49	24.89	Fruits
Rutin	609.1455 [M−H]	609.1440	4.28	2.46	Flowers
		609.1433	4.27	3.61	Fruits
		609.1439	4.37	2.63	Leaves
Quercimetrin	463.0876 [M−H]	463.0863	4.44	2.81	Flowers
Kaempferol 5-glucoside	447.0927 [M−H]	447.0916	4.61	2.46	Flowers
		447.0915	4.54	2.68	Fruits
Isorhamnetin 7-glucoside	477.1033 [M−H]	477.1014	4.61	3.98	Flowers
		477.1011	4.59	4.61	Fruits
Kaempferol-3-O-glucoside-7-O-rhamnoside	593.1506 [M−H]	593.1503	4.37	0.51	Flowers
		593.1482	4.42	4.05	Fruits
Kaempferol	285.0399 [M−H]	285.0395	5.49	1.40	Flowers
		285.0392	5.45	2.46	Fruits
Quercetin	301.0348 [M−H]	301.0347	5.17	0.33	Flowers
		301.0339	5.14	2.98	Fruits
Ascorbic acid	175.0237 [M−H]	175.0240	2.28	1.71	Flowers
		175.0236	2.26	0.57	Fruits
4-hydroxybenzoic acid	137.0233 [M−H]	137.0239	5.55	4.37	Flowers
		137.0237	5.54	2.91	Fruits

RT (Retention Time).

**Table 4:** Cellular antioxidant activity (CAA) of *Gallesia integrifolia* flowers’, leaves’, and fruits’ essential oil and crude extract.

Samples	Inhibition at the Maximum Concentration Tested (%)
Leaves EO	82 b
Fruits EO	82 b
Flowers EO	69 e
Leaves CE	77 d
Fruits CE	81 c
Flowers CE	81 c
Quercetin	93 a

Maximum concentration tested (2000 µg/mL). Quercetin was used as positive control and was tested at 0.3 g/mL. EO: essential oil; CE: crude extract. Inhibition at the maximum concentration tested (%); different letters in column differ significantly by Tukey’s HSD test (*p* ≤ 0.05).

**Table 5:** Antiproliferative activity (GI50 µg/mL) and selectivity index (SI) of *Gallesia integrifolia* flowers’, leaves’, and fruits’ essential oil and crude extract.

Samples	AGS	IS	Caco-2	IS	MCF-7	IS	NCI-H460	IS	Vero
Leaves EO	204.00 ± 4.00 bE	1.01	82.00 ± 3.00 aC	2.52	200.00 ± 17.00 bC	1.03	218.00 ± 12.00 bC	0.94	207.00 ± 11.00 bC
Fruits EO	113.00 ± 11.00 aC	2.00	88.00 ± 5.00 aC	2.57	209.00 ± 16.00 bC	1.08	221.00 ± 13.00 bC	1.02	226.00 ± 21.00 bC
Flowers EO	178.0 ± 18.0 bD	1.19	76.00 ± 5.00 aC	2.79	230.0 ± 23.0 cC	0.92	209.00 ± 11.00 bcC	1.01	212.00 ± 9.00 bcC
Leaves CE	68.00 ± 4.00 aB	2.53	51.00 ± 1.00 aB	3.37	138.00 ± 9.00 bB	1.24	222.00 ± 4.00 dC	0.77	172.00 ± 15.00 cB
Fruits CE	70.00 ± 4.00 aB	2.93	83.00 ± 2.00 aC	2.46	193.00 ± 5.00 bC	1.06	210.00 ± 8.00 cC	1.25	202.00 ± 3.00 bcBC
Flowers CE	81.33 ± 2.31 aB	2.52	144.00 ± 14.00 bD	1.42	209.00 ± 8.00 cC	0.98	164.00 ± 14.00 bB	1.25	205.00 ± 14.00 cBC
Ellipticine	1.23 ± 0.030 bA	1.15	1.21 ± 0.200 bA	1.16	1.02 ± 0.02 aA	1.38	1.010 ± 0.010 aA	1.39	1.41 ± 0.06 aA

The arithmetical averages with standard deviation followed by different uppercase letters in columns and lowercase letters in lines differ significantly by Tukey’s HSD test (*p* ≤ 0.05). GI₅₀ = concentration that causes 50% growth inhibition; SI = selectivity index. Ellipticine was used as positive control; AGS (gastric adenocarcinoma), Caco-2 (colorectal adenocarcinoma), MCF7 (breast adenocarcinoma), NCI-H460 (lung carcinoma), VERO (African green monkey kidney epithelial cells). EO: essential oil; CE: crude extract.

**Table 6:** Anti-inflammatory activity of *Gallesia integrifolia* flowers’, leaves’, and fruits’ essential oil and crude extract.

Samples	IC_50_ (µg/mL)
Leaves EO	54.00 ± 1.0 cb
Fruits EO	36.00 ± 3.00 b
Flowers EO	59.00 ± 4.00 c
Leaves CE	268.0 ± 21.00 d
Fruits CE	48.00 ± 2.00 cb
Flowers CE	51.00 ± 1.0 cb
Dexamethasone	6.300 ± 0.4 a

The arithmetical averages with the same letters do not differ significantly by Tukey’s HSD test (*p* ≤ 0.05). IC_50_ = effective concentration providing 50% inhibition of nitric oxide production. Dexamethasone was used as positive control. EO: essential oil; CE: crude extract.

## Data Availability

The datasets generated during and/or analyzed during the current study are available from the corresponding author on reasonable request.

## Sample Availability

Samples of the essential oils and crude extracts of leaves, flowers and fruits of *Gallesia integrifolia* are available from the authors.
